# High risk does not guarantee high accuracy—Evaluating the prognostic accuracy of OCT biomarkers for predicting late AMD

**DOI:** 10.1111/opo.13547

**Published:** 2025-06-25

**Authors:** Matt Trinh, Rene Cheung, Judy Nam, David Ng, Lisa Nivison‐Smith, Angelica Ly

**Affiliations:** ^1^ School of Optometry and Vision Science, University of New South Wales Sydney New South Wales Australia; ^2^ Centre for Eye Health Sydney New South Wales Australia; ^3^ Vision Eye Institute Sydney New South Wales Australia

**Keywords:** accuracy, age‐related macular degeneration, area under the receiver operating characteristic curve, biomarker, OCT, prognosis, sensitivity, specificity

## Abstract

**Purpose:**

The translation of high‐risk biomarkers into accurate predictions of late age‐related macular degeneration (AMD) may be limited by biomarker prevalence, subjective identification and competing risks from concurrent biomarkers. This study evaluates the prognostic performance of key optical coherence tomography (OCT) biomarkers for progression to late AMD, with colour fundus photography (CFP) as the reference standard.

**Methods:**

This retrospective study included 78 single eyes with intermediate AMD, propensity‐score matched by age and sex between converters and non‐converters to late AMD. Ten OCT biomarkers empirically derived from recent meta‐analysis, alongside CFP biomarkers of large drusen and pigmentary abnormality, were independently graded by three clinician‐researchers. Biomarkers' associated risk (odds ratios) and prognostic performance (area under the receiver operating characteristic curve (AUC), sensitivity, specificity) were evaluated for predicting late AMD.

**Results:**

The adjusted risk was highest for OCT‐detected nascent geographic atrophy, shallow irregular retinal pigment epithelium (RPE) elevations, drusenoid pigment epithelium detachment and RPE reflective abnormality (odds ratios, 6.66 [1.32, 42.71] to 28.27 [2.44, 545.3], *p* < 0.05).

However, CFP‐detected pigmentary abnormalities demonstrated the highest individual prognostic accuracy (77.69 [68.11, 87.27]% AUC, *p* < 0.0001), with excellent sensitivity (92.31%) but moderate specificity (63.08%). Adding at least three OCT biomarkers was required to improve prognostic performance significantly (91.01 [84.66, 97.36]%, *p* < 0.0001), and at least eight additional biomarkers to yield both excellent sensitivity (94.62%) and specificity (90.77%).

**Conclusions:**

CFP‐detected pigmentary abnormality remains a mainstay of clinical AMD prognostication, likely due to its higher prevalence and interpretability than high‐risk OCT biomarkers. Integrating OCT biomarkers into clinical prognostic models is promising but complex and may require automated identification to aid efficiency.

**Key points**

- Pigmentary changes seen on standard colour fundus photographs remain the most accurate individual feature for predicting progression in age‐related macular degeneration.
- High‐risk features seen on optical coherence tomography scans are linked to age‐related macular degeneration progression but often occur infrequently, too late or are difficult to grade reliably on their own.
- Combining multiple imaging features improves prediction accuracy for age‐related macular degeneration, but clinical use may be limited without automated tools to manage complexity and ensure consistency.

## INTRODUCTION

Accurate prognosis in age‐related macular degeneration (AMD) involves discriminating between patients at high‐ versus low‐risk of progressing to late AMD. This distinction helps prioritise timely and targeted care for higher risk individuals, including risk mitigation strategies such as dietary modifications and supplements,^1^ to minimise vision loss and maintain vision‐related quality of life.^2^

With the growing accessibility of optical coherence tomography (OCT),^3^ there has been increased focus on OCT biomarkers that may improve risk assessment for AMD progression.^4^ A recent meta‐analysis^4^ confirmed that several OCT biomarkers are associated with higher risks of progressing to late AMD relative to traditional colour fundus photography (CFP)‐detected large drusen, indicating potential for future integration into clinical prognostic models. However, strong risk associations (i.e., odds ratios and hazard ratios) may not necessarily translate into accurate predictions of disease progression.

Several factors may diminish the accuracy of OCT biomarkers for predicting late AMD. For example, biomarkers such as nascent geographic atrophy (nGA), while serving as an immediate high‐risk precursor lesion for GA,^5^, ^6^ may under‐perform in predictive accuracy due to its late appearance relative to other biomarkers. This may be further compounded by low biomarker prevalence,^7^, ^8^ subjective identification^9^ or competing risks from concurrent biomarkers,^10^ all of which may limit prognostic accuracy.^11^ Early identification of high‐risk cases using biomarkers with sufficient prevalence and predictive accuracy could provide valuable lead time to implement risk mitigation strategies and optimise visual outcomes.

This study focuses on evaluating biomarkers across a median follow‐up of approximately 3 years, extending up to 7 years, to reflect a pragmatic clinical horizon for risk stratification and early intervention. Ten empirically derived OCT biomarkers,^4^ selected from prior meta‐analysis, were assessed to determine whether their associated risks translate into accurate predictions of conversion to late AMD. Results will inform whether current OCT biomarkers are sufficiently accurate for clinical prognostication, enabling more timely and targeted care strategies to reduce the burden of vision loss in AMD.

## METHODS

### Study population

Consecutive patients with a clinical diagnosis of intermediate AMD at any visit and longitudinal follow‐up were sampled retrospectively from Centre for Eye Health (CFEH) records dated from 1 January 2009 (inception) to 31 December 2022. Eyes with concurrent, confirmed or suspected macular and/or optic nerve disease were excluded. The CFEH is an intermediary eyecare centre in Sydney, Australia, providing advanced and strictly standardised diagnostic testing and management of chronic eye diseases by optometrists and ophthalmologists. Imaging follow‐up intervals were standardised to a maximum of 12 months for patients with AMD. Background factors such as race/ethnicity and smoking status were collected routinely via entrance screening forms. All patients who had their records reviewed had provided written informed consent for use of their de‐identified data in accordance with the Biomedical Human Research Ethics Advisory Panel from the University of New South Wales and the Declaration of Helsinki.

Clinical diagnoses of intermediate AMD were formed by at least two unblinded clinicians and further confirmed by authors MT, RC and JN according to the Beckman Initiative eye‐level classification system,^12^ including eyes with large drusen or pigmentary abnormality with at least medium drusen. The sample size was calculated a priori, hypothesising that OCT biomarkers were associated with greater risk of conversion to late AMD than CFP‐detected large drusen. Assuming a 95% confidence level, a case‐to‐control ratio of 1:5 and an event prevalence of ≈20% in the non‐biomarker group (based on an internal audit of the CFEH population), the calculation incorporated an estimated odds ratio of 3.6. This odds ratio was derived from a pooled estimate^4^ of OCT studies evaluating risk associated with CFP‐identified large drusen.^13^, ^14^, ^15^ This yielded a required total sample size of *n* = 78.

Accordingly, 13 converters were randomly selected from consecutive patients with late AMD in either eye, with the first eye to develop late AMD (macular‐involving neovascularisation and/or geographic atrophy) included to minimise confounding from fellow‐eye risk. These converter eyes were propensity score matched by age and sex to 65 non‐converters, randomly selected from consecutive patients with no incidence of late AMD in either eye, with one eye per person randomly chosen for analysis. Fuzzy matching of propensity scores without replacement was used to maximise sample size and reduce the possibility of over‐matching in a limited pool of patients. Match tolerance was increased after each iterative random draw until a total *n* = 78 was reached. This statistical procedure helped to mitigate the imbalance of potential confounders between groups, while setting a minimal sample size reduced the possibility of incurring type I errors.

### Imaging biomarkers

**FIGURE 1:**
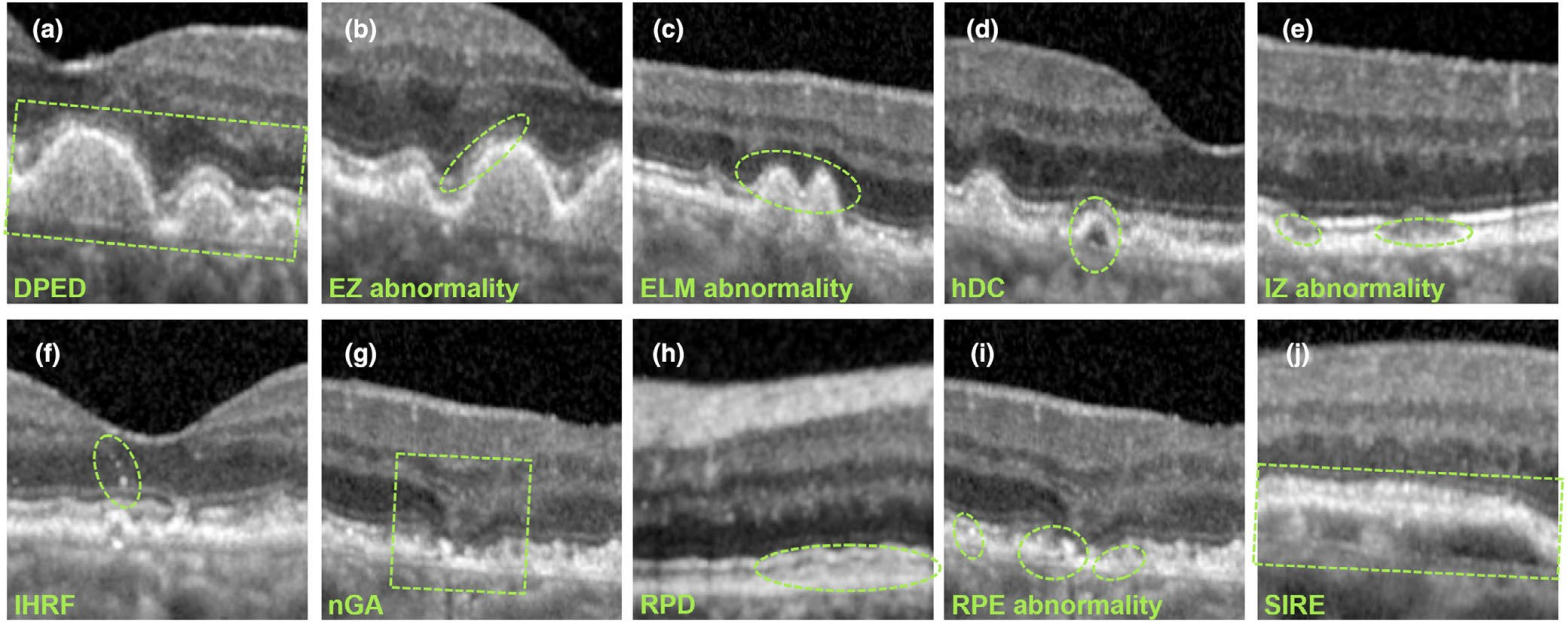
Representative examples of the Optical Coherence Tomography (OCT) biomarkers. (a) Drusenoid pigment epithelium detachment (DPED)—‘…at least 350 μm in the narrowest diameter…’; (b) ellipsoid zone (EZ) reflective abnormality—the second outer retinal hyper‐reflective band; (c) external limiting membrane (ELM) reflective abnormality—the first outer retinal hyper‐reflective band; (d) hypo‐reflective drusen cores (hDC)—‘…more hyporeflective than a typical druse…’; (e) interdigitation zone (IZ) reflective abnormality—the third outer retinal hyper‐reflective band; (f) intra‐retinal hyper‐reflective foci (IHRF)—‘…discrete…with greater reflectivity than the RPE band…’; (g) nascent geographic atrophy (nGA)—‘…subsidence of the outer plexiform layer and inner nuclear layer…or…hyporeflective wedge‐shaped band…’; (h) reticular pseudodrusen (RPD)—‘…≥5 definite lesions seen on >1 OCT B‐scan…’ above the RPE, (i) retinal pigment epithelium (RPE) reflective abnormality—the fourth outer retinal hyper‐reflective band and (j) shallow irregular RPE elevations (SIRE)—‘…RPE elevation…more than 1000 μm [width]…[height] less than 100 μm…and non‐homogeneous internal reflectivity…’, outlined in green. Full definitions provided verbatim in Table S1.

Ten OCT biomarkers were identified within OCT 30 × 25° 61 B‐scan images (Spectralis SD‐OCT, Heidelberg Engineering; heidelbergengineering.com), empirically derived from recent meta‐analysis.^4^ These biomarkers were selected following rigorous application of the Grading of Recommendations, Assessment, Development and Evaluation (GRADE) framework, comprising biomarkers with either ‘high certainty of evidence’ or those meeting all but one criterion, typically due to limited studies reflecting their recent emergence in the literature. These 10 biomarkers included drusenoid pigment epithelium detachment (DPED), ellipsoid zone (EZ) abnormality, external limiting membrane (ELM) abnormality, hypo‐reflective drusen cores (hDC), interdigitation zone (IZ) abnormality, intra‐retinal hyper‐reflective foci (IHRF), nascent geographic atrophy (nGA), reticular pseudodrusen (RPD), retinal pigment epithelium (RPE) abnormality and shallow irregular RPE elevation (SIRE). Representative examples are depicted in Figure 1, with exact definitions provided in Table S1.

Two CFP biomarkers were identified using CFP 30° posterior pole images (VK‐2 fundus camera, Kowa, ophthalmic.kowa‐usa.com), derived from the simplified Age‐Related Eye Disease Study (AREDS) scale.^16^ These included large drusen and pigmentary abnormality (Table S1).

### Biomarker grading

Grading protocol was defined a priori, whereby three independent graders, blinded to participant's converter/non‐converter status, assessed each biomarker's presence in the relevant OCT or CFP scans according to a trinary system of certainty (≥90% certain, 50%–90% certain or <50% certain). If the presence of a biomarker was graded as 50%–90% certain, an auxiliary imaging modality was selected by each grader to supplement grading, to replicate multimodal imaging in clinical practice. Grading continued until each biomarker was deemed present (≥90% certainty) or absent (<50% certainty), or until there were no more imaging modalities to supplement grading. To set a high threshold of certainty, a final grading of 50%–90% certain was also considered equivalent to the biomarker being ‘absent’, consistent with previous methodologies.^17^ The final grading for each biomarker was determined by majority agreement.

### Outcomes

Preliminary outcomes included biomarkers' prevalence, inter‐grader reliability and median time‐to‐conversion. The main outcome was biomarkers' risk associations (odds ratios) and prognostic accuracy (AUC and precision‐recall PR‐AUC) for predicting late AMD for individual and combined biomarkers.

### Statistical analysis

Statistical analyses were performed using Prism 10.1 (GraphPad; graphpad.com/), IBM SPSS 27 (ibm.com/spss), Microsoft Excel 2205 (microsoft.com/excel) and Python 3.12.6 (Python Software Foundation; python.org/). Default statistical significance was *p* < 0.05. Summary continuous values were presented as mean [95% confidence intervals (CI)] unless specified otherwise. Comparison of continuous data between groups was performed using student's *t‐*test and of categorical data between groups using Fisher's exact test. Prevalence was reported as a percentage for converters and non‐converters to late AMD. Inter‐grader reliability was reported as overall agreement and free‐marginal *κ*; the latter interpreted as minimal (0.21–0.39), weak (0.4–0.59), moderate (0.6–0.79), strong (0.8–0.9) and almost perfect (>0.9).^18^ Median time‐to‐conversion significance was calculated using the log‐rank (*χ* ^2^) test.

Adjusted risk considering all biomarkers was calculated using the odds ratio (rather than time‐dependent hazard ratios), employing stepwise backward elimination (removing the variable with the highest *p*‐value) and forward selection (adding the variable with the lowest *p*‐value) until all variables reached *p* < 0.10.^19^ Prognostic performance was calculated using area under the receiver operating characteristic curve (AUC) with parametric CIs,^20^ applying stepwise backward and forward selection based on the AUC. Given the imbalance between converters (*n* = 13) and non‐converters (*n* = 65), precision‐recall (PR)‐AUC was calculated additionally using bootstrapping (*n* = 1000) to estimate CIs and empirical statistical significance relative to the outcome prevalence (≈17%).^21^ Due to limited sample size, internal validation through data splitting (e.g., training–test–validation) was not performed and analyses were conducted on the full data set. This approach carries a risk of optimism bias^22^; however, biomarker selection was guided by prior meta‐analytic evidence to reduce overfitting. Findings should nonetheless be interpreted as exploratory and hypothesis‐generating, pending external validation. AUC was interpreted as a fail (≥50%–60%), poor (≥60%–70%), fair (≥70%–80%), good (≥80%–90%) or excellent (≥90%).^23^ Given PR‐AUC's dependence on outcome prevalence, values were expectedly lower than the AUC and should be contextualised against the baseline prevalence of the positive class, rather than Reciever Operating Characteristic (ROC)‐derived thresholds.^21^ These variable selection methods were utilised rather than other popular methods such as Least Absolute Shrinkage and Selection Operator and tree‐based, to maintain parsimony in a relatively small data set.^24^ Further evaluation of prognostic performance was performed using Youden's Index to calculate optimal sensitivity and specificity.^25^ Prognostic performance was compared between models using DeLong's method.^26^

## RESULTS

### Study population

Overall, participants were 71.86 [70.18, 73.54] years of age, 56.41% female, 50% White race/ethnicity, 20.51% had ever smoked and follow‐up time was 2.74 years on average and extended up to 7.27 years. There were no significant differences between the converter (*n* = 13) and non‐converter (*n* = 65) groups for any of the above factors (*p* = 0.34–0.8; Table 1).

**TABLE 1:** Study population characteristics by group.

	Converters	Non‐converters	*p‐*value	Total
Age (years)	73.01 [67.28, 78.74]	71.63 [69.88, 73.37]	0.54	71.86 [70.18, 73.54]
Sex (female: male)	6:7	38:27	0.54	44:34
Race/ethnicity (White:Asian:Other)	5:0:8	34:4:27	0.34	39:4:35
Smoking status (ever:never)	3:10	13:52	0.80	16:62
Follow‐up time (years)	2.85 [1.73, 3.97]	2.72 [2.23, 3.22]	0.60	2.74 [2.3, 3.19]

*Note*: *p*‐value refers to converters versus non‐converters. Note that from the converters, 61.5% progressed to geographic atrophy and 38.5% progressed to neovascularisation.

### Prevalence, inter‐grader reliability and time‐to‐conversion

Biomarkers' prevalence, inter‐grader reliability and time‐to‐conversion were examined to provide context for their associated risks and prognostic performance. Biomarkers' prevalence ranged between 62%–100% in converters and 3%–91% in non‐converters. IZ reflective abnormality and large drusen demonstrated the highest prevalence and were present in most converter and non‐converter eyes (85%+; Table S2). NGA and SIRE demonstrated the lowest prevalence and were present in 46% and 38% of converter eyes, respectively, but only 3% of non‐converter eyes.

Inter‐grader reliability ranged from minimal (ELM reflective abnormality, 62.39% agreement and 0.25 [0.1, 0.4] *κ*) to moderate (nGA, 88.03% agreement and 0.76 [0.37, 0.87] *κ*; Table S2).

The median time‐to‐conversion was significant for IHRF, pigmentary abnormality, hDC, RPE reflective abnormality, DPED, nGA and SIRE (2.74–4.81 years, *p* < 0.05; Table S2). The presence of any other biomarker was not significantly associated with conversion in this study population.

### Associated risk of conversion to late AMD

Subsequently, biomarkers' associated risk of conversion to late AMD was evaluated as unadjusted odds ratios. In descending order, nGA, pigmentary abnormality, SIRE, IHRF, DPED, RPE reflective abnormality and hDC were significantly associated with conversion to late AMD (3.84 [1.23, 15.45] to 27 [5.17, 211.2], *p* < 0.05; Table 2).

**TABLE 2:** Biomarkers' associated risk of conversion to late AMD.

Biomarker	Unadjusted odds ratio	Adjusted odds ratio
nGA	27 [5.17, 211.2]***	28.27 [2.44, 545.3]*
Pigmentary abnormality	20.5 [3.69, 385.1]**	**–**
SIRE	19.69 [3.62, 154.9]**	11.75 [1.01, 173.1]*
IHRF	14.9 [2.7, 279.3]*	**–**
DPED	8.2 [2.32, 34.12]**	6.66 [1.32, 42.71]*
RPE reflective abnormality	4.52 [1.33, 16.81]*	8.55 [1.5, 79.74]*
hDC	3.84 [1.23, 15.45]*	**−**

*Note*: Biomarkers presented in descending order of unadjusted odds ratio. Significant values bolded, *p < 0.05, **p < 0.01, ***p < 0.001; non‐significant values denoted by ‘–’. Other biomarkers not listed were non‐significant for unadjusted or adjusted odds ratios, that is, ELM reflective abnormality, EZ reflective abnormality, IZ reflective abnormality, large drusen and RPD.

Abbreviations: DPED, drusenoid pigment epithelium detachment; ELM, external limiting membrane; EZ, ellipsoid zone; hDC, hypo‐reflective drusen cores; IHRF, intra‐retinal hyper‐reflective foci; IZ, interdigitation zone; nGA, nascent geographic atrophy; RPD, reticular pseudodrusen; RPE, retinal pigment epithelium; SIRE, shallow irregular RPE elevations.

Adjusted for competing risks (i.e., concurrent presence of other biomarkers), significant risk remained for nGA (28.27 [2.44, 545.3], *p <* 0.05), SIRE (11.75 [1.01, 173.1], *p <* 0.05), DPED (6.66 [1.32, 42.71], *p <* 0.05) and RPE reflective abnormality (8.55 [1.5, 79.74], *p <* 0.05; Table 2), with large variability.

### Prognostic performance for conversion to late AMD

Biomarker's prognostic performance was then assessed, with individual biomarkers' prognostic accuracy (AUC) ranging from fail (IZ abnormality, 54.6 [51.1, 58.2]%, *p <* 0.05) to fair (pigmentary abnormality, 77.7 [68.1, 87.3]%, *p <* 0.0001; Table S3). When considering PR‐AUC, values were expectedly lower and ranged between 45.9 [27.6, 60.8]% (*p <* 0.01) and 65.1 [36.3, 84] (*p <* 0.0001), with nGA and pigmentary abnormalities demonstrating the highest performance (Table S3). CFP‐detected pigmentary abnormalities consistently demonstrated the highest (AUC) or second highest (PR‐AUC) prognostic accuracy for any single biomarker (77.7 [68.1, 87.3]% AUC, *p <* 0.0001; 63.6 [49.7, 73.5]% PR‐AUC, *p <* 0.0001) with excellent sensitivity (92.31%), albeit moderate specificity (63.08%; Table S4). Accordingly, pigmentary abnormality was used as the reference standard for the following analyses.

**FIGURE 2:**
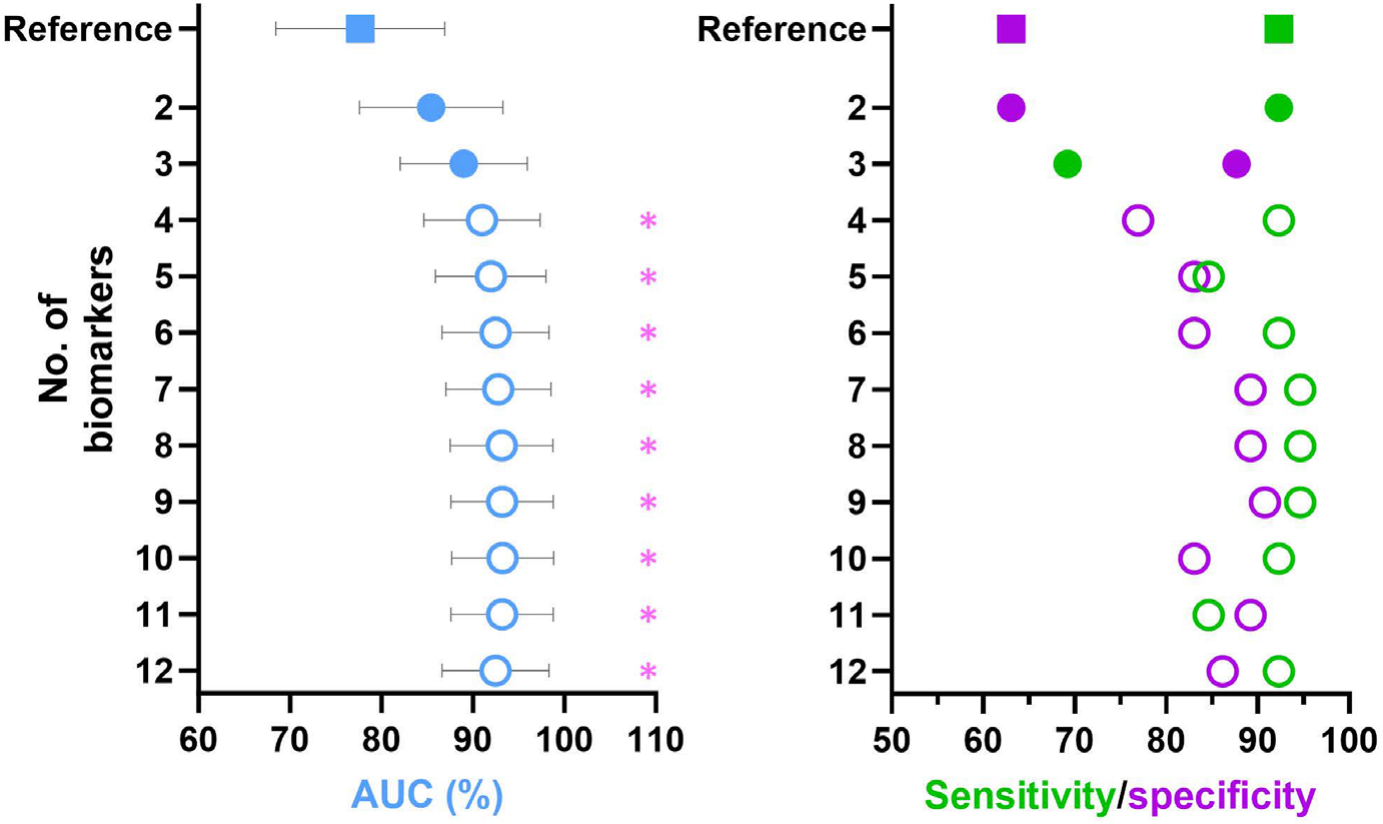
Biomarkers' combined prognostic accuracy for conversion to late age‐related macular degeneration (AMD). Note the trend towards increased area under the receiver operating characteristic curve (AUC) [CI]^20^ (blue) and sensitivity (green)/specificity (purple) with the addition of each biomarker. Starting from the reference model using pigmentary abnormality alone (*squares*), one biomarker was added at a time (*filled circles*) using stepwise backward and forward selection. A significant improvement was only evident from model no. 4 onwards, with at least three added optical coherence tomography (OCT) biomarkers (*open circles*). Excellent (≥90%) sensitivity and specificity were reached from model no. 9 onwards, with at least eight additional biomarkers. Significant values bolded *p < 0.05. Exact models including precision‐recall area under the curve (PR‐AUC) are described in Table S4.

Combining OCT and CFP biomarkers showed a trend towards improved AUC, PR‐AUC, (Figure 2a) and sensitivity/specificity (Figure 2b), relative to pigmentary abnormality alone.

A minimum of three added OCT biomarkers (DPED, SIRE and RPE reflective abnormalities; Table S4) were required to produce a significant improvement, whereby prognostic accuracy was excellent (91 [84.7, 97.4]% AUC, *p <* 0.05; 75.1 [50.7, 92.1]% PR‐AUC), sensitivity was excellent (92.31%) and specificity was fair (76.92%; Figure 2a,b).

A minimum of eight added biomarkers (SIRE, RPE reflective abnormalities, EZ reflective abnormalities, nGA, DPED, hDC, IHRF and large drusen; Table S4) were required to yield excellent (≥90%) sensitivity and specificity (93.2 [87.6, 98.8]% AUC, *p <* 0.05; 79.3 [55.2, 95.1] PR‐AUC; sensitivity 94.62%, specificity 90.77%). Subsequent models with additional biomarkers showed incremental changes.

## DISCUSSION

This study evaluated the translation of high‐risk OCT biomarkers into prognostic performance for conversion to late AMD. While the adjusted risk was highest for OCT‐detected nGA, SIRE, DPED and RPE reflective abnormality, CFP‐detected pigmentary abnormality demonstrated the highest individual prognostic accuracy. Adding at least three OCT biomarkers was required to improve prognostic performance significantly beyond using pigmentary abnormality alone, and at least eight additional biomarkers were required to yield both excellent sensitivity and specificity. Thus, CFP‐detected pigmentary abnormality remains a mainstay of clinical AMD prognostication, likely due to its higher prevalence and interpretability. Integrating OCT biomarkers into clinical prognostic models is promising but complex and may require automated identification to aid efficiency.

### Prevalence, inter‐grader reliability and time‐to‐conversion may hinder some OCT biomarkers' clinical usage

This study contextualised the prevalence and inter‐grader reliability of key OCT biomarkers, which were generally consistent with previous reports.^27^ Although biomarkers such as nGA and SIRE were strongly associated with progression to late AMD, their relatively lower prevalence (Table S2) likely limited their prognostic utility. As such, high‐risk but less common biomarkers may not be as useful in future imaging‐based prognostic models unless supported by more common biomarkers like RPD.^27^, ^28^, ^29^ Large drusen also lacked discriminatory power, though likely because they were nearly ubiquitous in eyes with intermediate AMD. Meanwhile, pigmentary abnormality emerged as the single most useful predictor of disease progression, combining moderate prevalence in both converter and non‐converter eyes with strong discriminatory performance. This aligns with prior findings, including foundational data that identified pigmentary abnormality as a significant predictor of progression,^16^ and further bolstered by our recent systematic review describing IHRF, an OCT sign which heavily overlaps with pigmentary abnormality, to be associated with one of the highest independent risks of conversion to late AMD.

Furthermore, the inter‐grader reliability of biomarkers was comparable to several multi‐biomarker OCT studies,^9^, ^30^, ^31^ though slightly lower than some other reports,^32^, ^33^, ^34^ possibly due to the use of optometrist‐researchers for grading rather than specialised reading‐centre personnel. Nevertheless, this approach mirrors real‐world clinical settings, where optometrists or general medical practitioners often encounter intermediate AMD first and then triage patients for further monitoring or referral. It also underscores the need for biomarkers that non‐specialists can identify reliably to maximise downstream prognostic outcomes, or alternatively, the development of biomarkers that can be identified objectively and automatically, for example, using artificial intelligence.

Assessment of time‐to‐conversion complemented prevalence and risk estimates, providing insight into biomarkers' clinical relevance. Although nGA and SIRE were less prevalent, their relatively short time‐to‐conversion (2.7 years) indicates proximity to progression but a limited window for early intervention. In contrast, IHRF and pigmentary abnormality combined moderate time‐to‐conversion (3.35–3.61 years) with higher prevalence, enhancing their utility for early risk stratification. Conversely, highly prevalent biomarkers such as large drusen and EZ reflective abnormality did not demonstrate significant associations with time‐to‐conversion within the 7‐year follow‐up, thereby limiting their prognostic utility in isolation. Collectively, these findings underscore that clinically meaningful biomarkers require not only robust risk association but also sufficient prevalence and timely occurrence, supporting the use of multi‐biomarker strategies for improved prognostication.

### Integrating OCT biomarkers for clinical AMD prognostication

Other OCT‐inclusive prognostic models have been proposed,^35^, ^36^, ^37^, ^38^, ^39^, ^40^, ^41^ but have been derived ad hoc (rather than defined a priori from systematic evidence)^4^ and hence prone to selection bias and under‐representation of the competing risks associated with concurrent OCT biomarkers.^35^, ^37^, ^38^, ^39^, ^40^, ^42^ Table 3 summarises the various OCT biomarkers which have been assessed in the literature, primarily using artificial intelligence, showing AUCs between 75% and 96% and comparable to this study.

**TABLE 3:** Alternate optical coherence tomography (OCT)‐inclusive models of age‐related macular degeneration (AMD) prognostication.

Study	DPED	Drusen morphology	Drusen reflectivity	EZ Abn	IHRF	iRORA	Retinal morphology	RPD	AUC (%)	Follow‐up time
Dow et al. 2023^42^, ^b^	a				a		a	a	88	2 years
Flores et al. 2023^36^		a		a	a	a			77	2 years
Sarici et al. 2022^37^, ^b^	a			a			a	a	96	2 years
Schmidt‐Erfurth et al. 2018^40^, ^b^		a			a		a	a	80	2 years
de Sisternes et al. 2014^38^, ^b^		a	a						79	4 years
Wu et al. 2021^35^, ^b^		a	a		a		a		88	3 years
Yim et al. 2020^39^, ^b^		a			a				75	0.5 years

*Note*: The current study's prognostic models approached 93% AUC. Note that other studies used varying conditions, and for simplicity, the highest AUC has been reported for follow‐up times closest to 3 years.

Abbreviations: Abn, abnormality; AUC, area under the receiver operating characteristic curve; DPED, drusenoid pigment epithelial detachment; EZ, ellipsoid zone; IHRF, intra‐retinal hyper‐reflective foci; iRORA, incomplete retinal pigment epithelium and outer retinal atrophy; RPD, reticular pseudodrusen.

^a^ Denotes that the OCT biomarker was used in prognostic modelling.

^b^ Denotes studies that used artificial intelligence algorithms, that is, all studies except Flores et al.^36^ ‘Morphology’ refers to size and/or shape, for example, height, width, thickness, volume, etc. Dow et al. also assessed other predictors including choroidal morphology, vitreomacular abnormalities, cystoid macular oedema, sub‐retinal fluid, vitelliform lesion and other features.^42^

To our knowledge, these OCT‐based models have not been compared directly against the easy‐to‐use simplified AREDS risk scale,^16^, ^29^ based on CFP detection of drusen size, pigmentary abnormalities^43^ and more recently, RPD.^29^ In this study, we observed that integrating OCT biomarkers, though promising, can be inefficient. As expected, PR‐AUC values were lower than (ROC) AUC values, reflecting their heightened sensitivity to group imbalance; however, both metrics demonstrated relatively concordant trends across individual biomarkers and incremental improvements with the addition of OCT biomarkers. At least three additional biomarkers were required to enhance prognostic performance beyond using CFP‐detected pigmentary abnormality alone, and at least eight biomarkers were needed to reach ≥90% sensitivity and specificity. These findings emphasise the practical challenges of interpreting multiple OCT biomarkers in clinical settings. While advances in OCT interpretation and artificial intelligence are bridging this gap, the higher prevalence and interpretability of CFP‐based markers (relative to OCT markers) ensure that the simplified AREDS risk scale still remains a mainstay for clinical AMD prognostication.^16^, ^29^

These findings align with recent efforts to refine risk prediction, such as the PINNACLE trial,^44^ which is developing artificial intelligence‐driven, multimodal models incorporating imaging and genetic data for intermediate AMD. Similarly, Goh et al.^45^ demonstrated that although multimodal imaging modestly improved clinician predictions over CFP alone, a basic model comprising age, pigmentary abnormality and drusen volume outperformed both approaches, reinforcing the value of streamlined models. Collectively, these studies, together with the present findings, underscore the ongoing challenges in integrating complex biomarker data into clinical practice, and the need for automated, validated tools to enable scalable and practical risk stratification.

### Limitations

The primary limitation of this study was the relatively small sample size, drawn retrospectively from a single intermediary eyecare centre and a single OCT device, which may have restricted detection of biomarkers requiring extended follow‐up to establish prognostic significance. Consequently, the generalisability of findings to other clinical settings and OCT platforms could not be assessed. Although follow‐up intervals were standardised to a maximum of 12 months, the retrospective design introduced unavoidable interval‐censoring, reducing the precision of event timing and necessitating the use of odds ratios rather than hazard ratios for risk estimation.

Furthermore, internal validation through data splitting or cross‐validation was not performed due to sample size constraints, heightening the risk of overfitting during biomarker selection. Although biomarker selection was guided a priori by meta‐analytic evidence to mitigate this risk,^4^ external validation in larger, multi‐centre cohorts remains essential. Nonetheless, the study achieved its prespecified sample size for successful propensity‐score matching and the use of a minimally sized, a priori defined cohort helped reduce the risk of type I errors from inappropriate sampling. External validation is currently underway in larger, multi‐centre eyecare settings to substantiate these findings and enhance their clinical applicability. This will include stratification by conversion to geographic atrophy versus neovascular AMD, recognising that distinct biomarkers are likely associated with each subtype.^4^

Finally, manual identification of biomarkers is time‐intensive and subject to inter‐grader variability, although automated image interpretation is anticipated to become increasingly widespread. Regardless of future advances in supervised and unsupervised methods of biomarker identification and risk quantification, these approaches will continue to require validation against expert consensus as the reference standard,^46^ ensuring the ongoing relevance of the present findings as a foundational data set for future automated systems.

## CONCLUSIONS

CFP‐detected pigmentary abnormality remains a mainstay of clinical AMD prognostication, likely due to its higher prevalence and interpretability than high‐risk OCT biomarkers. Integrating OCT biomarkers into clinical prognostic models is promising but complex and may require automated identification to aid efficiency.

## AUTHOR CONTRIBUTIONS

**Matt Trinh:** Conceptualization (lead); data curation (lead); formal analysis (lead); investigation (lead); methodology (lead); project administration (lead); resources (lead); software (lead); supervision (lead); validation (lead); visualization (lead); writing – original draft (lead). **Rene Cheung:** Conceptualization (supporting); data curation (supporting); formal analysis (supporting); investigation (supporting); methodology (supporting); software (supporting); validation (supporting). **Judy Nam:** Data curation (supporting); formal analysis (supporting); investigation (supporting); validation (supporting); visualization (supporting). **David Ng:** Visualization (supporting). **Lisa Nivison‐Smith:** Conceptualization (supporting); funding acquisition (supporting); methodology (supporting); project administration (supporting); resources (supporting); supervision (supporting); visualization (supporting). **Angelica Ly:** Conceptualization (supporting); funding acquisition (equal); investigation (supporting); methodology (supporting); project administration (equal); resources (supporting); supervision (supporting); validation (supporting); visualization (supporting).

## CONFLICT OF INTEREST STATEMENT

The authors declare no commercial/competing relationships.

## MEETING PRESENTATIONS

Data from this manuscript was presented as an abstract poster at the 2024 Association for Research in Vision and Ophthalmology (ARVO) conference.

## Supporting information


Appendix S1.
