# Supplementary material for: High risk does not guarantee high accuracy—Evaluating the prognostic accuracy of OCT biomarkers for predicting late AMD
Source: Ophthalmic Physiol Opt. 2025 Jun 25;45(6):1293–301. doi: 10.1111/opo.13547 (PMC12357231; doi:10.1111/opo.13547)
Supplement: Supplementary file 1 — Appendix S1. [file OPO-45-1293-s001.docx]

# Supplementary material

High-risk does not guarantee high-accuracy –

Evaluating the prognostic accuracy of OCT biomarkers for predicting late AMD

Matt Trinh (PhD),^1^ Rene Cheung (PhD),^1^ Judy Nam (M Optom),^1^ David Ng (FRANZCO),^2^ *Lisa Nivison-Smith (PhD),^1^ *Angelica Ly (PhD)^1^

*^1^ School of Optometry and Vision Science, University of New South Wales, Sydney, NSW, Australia*

*^2^ Vision Eye Institute, Sydney, NSW, Australia*

**These authors contributed equally*

**Correspondence:** Dr Matt Trinh; [m.trinh@unsw.edu.au](mailto:m.trinh@unsw.edu.au)

School of Optometry and Vision Science, UNSW Sydney, 2015, NSW, Australia.

**S Table 1. Biomarkers’ definitions.**

Biomarkers were primarily identified using OCT or CFP only, with auxiliary viewing used only for ‘uncertain’ cases to replace usage of multimodal imaging in clinical practice.

CFP (*highlighted yellow*), colour fundus photography (VK-2 fundus camera, Kowa, USA); OCT, optical coherence tomography B-scans (Spectralis SD-OCT, Heidelberg Engineering, Germany); IR, *en face* infrared (Spectralis SD-OCT); OCT *en face* scans (Cirrus HD-OCT, Carl Zeiss Meditec, Germany), , and autofluorescence (Optomap, Daytona, USA). N/A, not applicable – refers to biomarkers without a commonly accepted definition using auxiliary imaging modality, e.g., abnormality of the outer retinal bands is exclusively an OCT B-scan biomarker.

| **Biomarker** | **Primary imaging modality definition** | **Auxiliary imaging modality definitions** |
| --- | --- | --- |
| **Drusenoid pigment epithelium detachment** | CFP: *see OCT* | OCT: “…well-defined, pale yellow or white, large mound consisting of many large drusen or confluent drusen which is at least 350 µm in the narrowest diameter and appears elevated…”^5^ |
| **Ellipsoid zone abnormality** | OCT: Continuous (homogeneous reflectivity), heterogeneous reflectivity, or discontinuous^6^ within central 1mm diameter | N/A |
| **External limiting membrane abnormality** | OCT: Continuous (homogeneous reflectivity), heterogeneous reflectivity, or discontinuous^6^ within central 1mm diameter | N/A |
| **Hypo-reflective drusen cores** | OCT: “…hyporeflective core…more hyporeflective than a typical druse…”^7^  *Associated with “…heterogenous internal reflectivity…and hyperreflective caps…calcified drusen”*^8^ | N/A |
| **Interdigitation zone abnormality** | OCT: Continuous (homogeneous reflectivity), heterogeneous reflectivity, or discontinuous^6^ within central 1mm diameter | N/A |
| **Intra-retinal hyper-reflective foci** | OCT: “…discrete, well-circumscribed lesions with greater reflectivity than the RPE band…”^9^ | CFP: *Pigmentary abnormality*^1^ *(see above definition)*  FAF: “…focal, linear, and/or lace-like increases in fundus autofluorescence…[excluding] reticular, focal plaque-like, or normal fundus autofluorescence patterns.”^2^  NIR: “Discrete, clumped deposits of intense hyper- or hypo-reflectivity”^3^  OCT *en face*: “Retinal slab (ILM to RPE)…seen as focal bright spots”^4^ |
| **Nascent geographic atrophy** | OCT: “…subsidence of the OPL and INL…or…hyporeflective wedge-shaped band…”^10^ | FAF: “…areas that were predominantly hyperautofluorescent…hypoautofluorescent…or mixed [most common]…”^11^  OCT *en face*: “Sub-RPE slab…showing areas of increased and decreased reflectivity.”^12^ |
| **Reticular pseudodrusen** | OCT: “≥ 5 definite [drusen-like] lesions seen on >1 OCT B-scan…” above the RPE.^13^  *Also referred to as sub-retinal drusenoid deposits.* | CFP: “…a network of faint interlacing ribbons”^14^ also referred to as branching^15^ or reticular^16^ pattern or “…more discrete dot-like deposits…”^14^ also referred to as discrete^15^ or dot^16^ pattern or “…moderately confluent globules in midperipheral regions.”^14^ also referred to as confluent pattern.^14,15^  FAF: “…center…isoautofluorescence, surrounded by a halo of reduced autofluorescence adjacent to the network of reticular autofluorescence…”^17^  NIR: “…center…isoreflectance, surrounded by a halo of reduced reflectance adjacent to the network of reticular IR reflectance…”^17^ |
| **Retinal pigment epithelium abnormality** | OCT: Continuous (homogeneous reflectivity), heterogeneous reflectivity, or discontinuous^6^ within central 1mm diameter | N/A |
| **Shallow irregular retinal pigment epithelium elevation** | OCT: “…RPE elevation…more than 1000µm…height…less than 100µm …(occasionally, small discrete sections could have slightly exceeded this cutoff)…and non-homogenous internal reflectivity…”^18^ | N/A |
| **Large drusen** | CFP: “…≥ 125µm in the smallest diameter…”^1^  “A distance approximating the width of a major branch retinal vein crossing the optic disc margin”^1^ | OCT: *See CFP* |
| **Pigmentary abnormality** | CFP: “Hyperpigmentation or hypopigmentation…in eyes with drusen 63µm or more in diameter and without known retinal disease entities or other reasons for such abnormality”^1^ | FAF: “…focal, linear, and/or lace-like increases in fundus autofluorescence…[excluding] reticular, focal plaque-like, or normal fundus autofluorescence patterns.”^2^  NIR: “Discrete, clumped deposits of intense hyper- or hypo-reflectivity”^3^  OCT: Hyperreflective foci^4^ (see below definition)  OCT *en face*: “Retinal slab (ILM to RPE)…seen as focal bright spots”^4^ |

**S Table 2. Biomarkers’ preliminary outcomes – prevalence, inter-grader reliability, and time-to-conversion.**

Biomarkers presented in descending order of overall prevalence. Significant values **bolded **P* < 0.05, ***P* < 0.01, *****P* < 0.0001;** non-significant values denoted by ‘**−**‘. † denotes the three highest values per statistic.

| **Biomarker** | **Prevalence**  **(converters)** | **Prevalence**  **(non-converters)** | **Inter-grader reliability**  **(agreement [%];**  **free-marginal κ)** | **Median time-to-conversion**  **(years)** | **Time-to-conversion *P* value** |
| --- | --- | --- | --- | --- | --- |
| **IZ reflective abnormality** | 100%† | 91%† | 77.78%;  0.56 [0.42, 0.7] | **−** | 0.21 |
| **Large drusen** | 100%† | 85%† | 82.05%;  0.64 [0.51, 0.77]† | **−** | 0.07 |
| **ELM reflective abnormality** | 85%† | 62% | 62.39%;  0.25 [0.1, 0.4] | **−** | 0.18 |
| **EZ reflective abnormality** | 92%† | 66%† | 66.67%;  0.33 [0.18, 0.48] | **−** | 0.14 |
| **IHRF** | 92%† | 45% | 70.09%;  0.4 [0.25, 0.55] | **3.61** | ******** |
| **Pigmentary abnormality** | 92%† | 37% | 66.67%;  0.33 [0.18, 0.48] | **3.35** | ******** |
| **hDC** | 69% | 37% | 67.52%;  0.35 [0.2, 0.5] | **3.35** | ****** |
| **RPD** | 62% | 42% | 68.38%;  0.37 [0.22, 0.52] | **−** | 0.11 |
| **RPE reflective abnormality** | 62% | 26% | 70.94%;  0.42 [0.27, 0.57] | **4.81** | ***** |
| **DPED** | 69% | 22% | 67.52%;  0.35 [0.2, 0.5] | **4.81** | ****** |
| **nGA** | 46% | 3% | 88.03%;  0.76 [0.37, 0.87]† | **2.74** | ******** |
| **SIRE** | 38% | 3% | 81.2%;  0.62 [0.49, 0.76] | **2.74** | ******** |

**S Table 3. Biomarkers’ individual prognostic accuracy for conversion to late AMD.**

Biomarkers presented in descending order of prognostic accuracy. Significant values **bolded **P* < 0.05, ***P* < 0.01, ****P* < 0.001**.

|  | **Prognostic accuracy (%)** | |
| --- | --- | --- |
| **Biomarker** | **AUC** | **PR-AUC** |
| **Pigmentary abnormality** | **77.7 [68.1, 87.3]****** | **63.6 [49.7, 73.5]****** |
| **IHRF** | **73.9 [64.2, 83.5]****** | **61.6 [48.7, 71]****** |
| **DPED** | **73.9 [59.9, 87.8]***** | **57.5 [38.1, 73.3]**** |
| **nGA** | **71.5 [57.3, 85.8]**** | **65.1 [36.3, 84]****** |
| **SIRE** | **67.7 [53.8, 81.6]*** | **60 [28.6, 80.4]**** |
| **RPE abnormality** | **67.7 [52.9, 82.5]*** | **50.5 [31.5, 68]**** |
| **hDC** | **66.2 [51.8, 80.5]*** | **50.9 [32.3, 65]****** |
| **EZ abnormality** | **63.1 [53.6, 72.6]**** | **58 [47, 65.8]****** |
| **ELM abnormality** | **61.5 [49.7, 73.4]** | **54.4 [40.5, 64.4]****** |
| **RPD** | 60 [45, 75] | 45.9 [27.6, 60.8]** |
| **Large drusen** | **57.7 [53.3, 62.1]***** | **59.7 [55.2, 64.6]****** |
| **IZ abnormality** | **54.6 [51.1, 58.2]*** | **59.1 [54.9, 63.8]****** |

**S Table 4. Biomarkers’ combined prognostic accuracy for conversion to late AMD.**

Note the trend toward increased AUC and sensitivity/specificity with the addition of each biomarker. A significant AUC improvement **(bolded **P* < 0.05)** was only evident from model no. 4 onwards, with at least 3 added OCT biomarkers, compared to the reference model: pigmentary abnormality alone. Significant values **bolded **P* < 0.05**. Models were formed using simultaneous backward and forward selection based on AUC.

Abn, abnormalities.

|  | **Prognostic accuracy (%)** | | **Sensitivity (%)** | **Specificity (%)** |
| --- | --- | --- | --- | --- |
| **No. of biomarkers** | **AUC** | **PR-AUC** |  |  |
| **Reference: Pigmentary abn** | 77.7  [68.1, 87.3] | 63.6  [49.7, 73.5] | 92.31 | 63.08 |
| **2: Pigmentary abn + DPED** | 85.4  [77.6, 93.3] | 62  [40.6, 80.6] | 92.31 | 63.08 |
| **3: Pigmentary abn + DPED + SIRE** | 89  [82.1, 95.9] | 73.4  [50.1, 90.6] | 69.23 | 87.69 |
| **4: Pigmentary abn + DPED + SIRE + RPE abn** | **91  [84.7, 97.4]*** | 75.1  [50.7, 92.1] | 92.31 | 76.92 |
| **5: Pigmentary abn + SIRE + RPE abn + EZ abn + nGA** | **92  [85.9, 98]*** | 74.3  [47.8, 91] | 84.62 | 83.08 |
| **6: Pigmentary abn + SIRE + RPE abn + EZ abn + nGA + DPED** | **92.5  [86.6, 98.3]*** | 79.6  [56.2, 95.2] | 92.31 | 83.08 |
| **7: Pigmentary abn + SIRE + RPE abn + EZ abn + nGA + DPED + hDC** | **92.8  [87, 87.5]*** | 79.9  [56.3, 95.3] | 94.62 | 89.23 |
| **8: Pigmentary abn + SIRE + RPE abn + EZ abn + nGA + DPED + hDC + IHRF** | **93.1  [87.5, 98.8]*** | 80.1  [56.3, 95.1] | 94.62 | 89.23 |
| **9: Pigmentary abn + SIRE + RPE abn + EZ abn + nGA + DPED + hDC + IHRF + large drusen** | **93.2  [87.6, 98.8]*** | 79.3  [55.2, 95.1] | 94.62 | 90.77 |
| **10: Pigmentary abn + SIRE + RPE abn + EZ abn + nGA + DPED + hDC + IHRF + large drusen + IZ abn** | **93.3  [87.7, 98.8]*** | 78.8  [54.9, 95] | 92.31 | 83.08 |
| **11: Pigmentary abn + SIRE + RPE abn + EZ abn + nGA + DPED + hDC + IHRF + large drusen + IZ abn + ELM abn** | **93.2  [87.6, 98.8]*** | 78.3  [54.1, 94.7] | 84.62 | 89.23 |
| **12: Pigmentary abn + SIRE + RPE abn + EZ abn + nGA + DPED + hDC + IHRF + large drusen + IZ abn + ELM abn + RPD** | **92.5  [86.6, 98.3]*** | 78.5  [54, 94.9] | 92.31 | 86.15 |

# Supplementary references

1. Ferris FL, Wilkinson CP, Bird A, et al. Clinical classification of age-related macular degeneration. *Ophthalmology*. 2013;120(4):844-851. doi:10.1016/j.ophtha.2012.10.036

2. Ly A, Nivison-Smith L, Assaad N, Kalloniatis M. Fundus autofluorescence in age-related macular degeneration. *Optom Vis Sci*. 2017;94(2):246-259. doi:10.1097/OPX.0000000000000997

3. Ly A, Nivison-Smith L, Assaad N, Kalloniatis M. Infrared reflectance imaging in age-related macular degeneration. *Ophthalmic Physiol Opt J Br Coll Ophthalmic Opt Optom*. 2016;36(3):303-316. doi:10.1111/opo.12283

4. Laiginhas R, Shi Y, Shen M, et al. Persistent hypertransmission defects detected on en face swept source optical computed tomography images predict the formation of geographic atrophy in age-related macular degeneration. *Am J Ophthalmol*. 2022;237:58-70. doi:10.1016/j.ajo.2021.11.001

5. Cukras C, Agrón E, Klein ML, et al. Natural history of drusenoid pigment epithelial detachment in age-related macular degeneration: AREDS report number 28. *Ophthalmology*. 2010;117(3):489-499. doi:10.1016/j.ophtha.2009.12.002

6. Ferrara D, Silver RE, Louzada RN, Novais EA, Collins GK, Seddon JM. Optical coherence tomography features preceding the onset of advanced age-related macular degeneration. *Invest Ophthalmol Vis Sci*. 2017;58(9):3519-3529. doi:10.1167/iovs.17-21696

7. Goh KL, Abbott CJ, Hadoux X, et al. Hyporeflective cores within drusen: association with progression of age-related macular degeneration and impact on visual sensitivity. *Ophthalmol Retina*. 2022;6(4):284-290. doi:10.1016/j.oret.2021.11.004

8. Liu J, Laiginhas R, Shen M, et al. Multimodal Imaging and En Face OCT Detection of Calcified Drusen in Eyes with Age-Related Macular Degeneration. *Ophthalmol Sci*. 2022;2(2):100162. doi:10.1016/j.xops.2022.100162

9. Ho J, Witkin AJ, Liu J, et al. Documentation of intraretinal retinal pigment epithelium migration via high-speed ultrahigh-resolution optical coherence tomography. *Ophthalmology*. 2011;118(4):687-693. doi:10.1016/j.ophtha.2010.08.010

10. Wu Z, Luu CD, Ayton LN, et al. Optical coherence tomography–defined changes preceding the development of drusen-associated atrophy in age-related macular degeneration. *Ophthalmology*. 2014;121(12):2415-2422. doi:10.1016/j.ophtha.2014.06.034

11. Wu Z, Luu CD, Ayton LN, et al. Fundus Autofluorescence Characteristics of Nascent Geographic Atrophy in Age-Related Macular Degeneration. *Invest Ophthalmol Vis Sci*. 2015;56(3):1546-1552. doi:10.1167/iovs.14-16211

12. Schaal KB, Gregori G, Rosenfeld PJ. En Face Optical Coherence Tomography Imaging for the Detection of Nascent Geographic Atrophy. *Am J Ophthalmol*. 2017;174:145-154. doi:10.1016/j.ajo.2016.11.002

13. Wu Z, Kumar H, Hodgson LAB, Guymer RH. Reticular pseudodrusen on the risk of progression in intermediate age-related macular degeneration. *Am J Ophthalmol*. 2022;239:202-211. doi:10.1016/j.ajo.2022.03.007

14. Suzuki M, Sato T, Spaide RF. Pseudodrusen subtypes as delineated by multimodal imaging of the fundus. *Am J Ophthalmol*. 2014;157(5):1005-1012. doi:10.1016/j.ajo.2014.01.025

15. Lee MY, Yoon J, Ham DI. Clinical features of reticular pseudodrusen according to the fundus distribution. *Br J Ophthalmol*. 2012;96(9):1222-1226. doi:10.1136/bjophthalmol-2011-301207

16. Zhou Q, Daniel E, Maguire MG, et al. Pseudodrusen and incidence of late age-related macular degeneration in fellow eyes in the comparison of age-related macular degeneration treatments trials. *Ophthalmology*. 2016;123(7):1530-1540. doi:10.1016/j.ophtha.2016.02.043

17. Querques G, Querques L, Martinelli D, et al. Pathologic insights from integrated imaging of reticular pseudodrusen in age-related macular degeneration. *Retina*. 2011;31:518-526. doi:10.1097/IAE.0b013e3181f04974

18. Narita C, Wu Z, Rosenfeld PJ, et al. Structural oct signs suggestive of subclinical nonexudative macular neovascularization in eyes with large drusen. *Ophthalmology*. 2020;127(5):637-647. doi:10.1016/j.ophtha.2019.11.007

19. Chew EY, Clemons TE, Agrón E, et al. Ten-year follow-up of age-related macular degeneration in the age-related eye disease study: AREDS report no. 36. *JAMA Ophthalmol*. 2014;132(3):272-277. doi:10.1001/jamaophthalmol.2013.6636
